# Effect of time of day and physical exercise on inflammatory biomarkers in athletic horses

**DOI:** 10.3389/fvets.2025.1608770

**Published:** 2025-06-04

**Authors:** Francesca Aragona, Claudia Giannetto, Giuseppe Piccione, Francesca Arfuso, Federica Arrigo, Antonino Costa, Salvatore De Caro, Antonio Cannuli, Francesco Fazio

**Affiliations:** ^1^Department of Veterinary Science, University of Messina, Messina, Italy; ^2^Department of Engineering, University of Messina, Messina, Italy

**Keywords:** horse, exercise, immune system, leukocyte subpopulation, inflammatory response, CD4-CD8, cytokine

## Abstract

**Introduction:**

The present study aimed to investigate the effect of time of day and physical exercise on some inflammatory biomarkers (white blood cell count-WBCs, leukocyte subpopulation CD4 + and CD8 + cells, total proteins, Interleukins IL-6, IL-1β and Tumor Necrosis Factor TNFα) in trained saddle horses.

**Methods:**

Blood samples were collected from 10 horses before, immediately (following 5 min) and 1 h after the training session in the morning (am) and afternoon (pm).

**Results and discussion:**

Statistical analysis showed increasing value of WBCs immediately and 1 h after exercise (*p* < 0.01: *p* < 0.01) at am compared to rest. Lower CD4 + oncentration was observed immediately and 1 h after exercise (*p* < 0.01; *p* < 0.01) at pm compared to rest and decreased levels 1-h post-exercise at pm compared to am (*p* < 0.001). CD8 + exhibited significant lower value at pm compared to am at each time point (before: *p* < 0.01; immediately: *p* < 0.01; 1 h after: *p* < 0.001). IL-6 showed increasing value post-exercise at pm (*p* < 0.01). IL-6 and IL-1β levels were markedly elevated at pm compared to am at each time point (*p* < 0.0001; *p* < 0.0001; *p* < 0.0001). This information is essential for formulating suitable training regimens, considering performance in connection to possible daily risk for infection, illness, and inflammation to which the horse may be vulnerable.

## 1 Introduction

Inflammation is defined by a sequence of cellular and molecular processes resulting in elevated body temperature, capillary dilatation, and the synthesis of soluble blood components in mammals (1, 2). These reactions, elicited by stress, are crucial for host defence and natural tissue homeostasis, initiating the elimination of harmful chemicals and damaged tissues (1, 3). Inflammation is a biological reaction of the immune system that prevents, restricts, and repairs damage caused by invading pathogens or endogenous biomolecules as observed in horses (4). In human medicine, acute inflammation is a temporary inflammatory response that benefits the organism, and a chronic inflammatory response is linked to tissue malfunction and pathology (5). Infection, physical trauma and exercise trigger the inflammatory response, which is controlled by the immune system. Physical exercise represents a stressor; it induces perturbations in physiological system, specifically affecting metabolic, circulatory, hormonal, and thermoregulatory pathways leading to the disruption of homeostasis.

This is followed by the activation of white blood cells and an increase in inflammatory mediators in the bloodstream, produced by immune cells and directly from active muscle tissue, to reestablish balance (6). Intense physical exercise stimulates the recruitment of lymphocytes into circulation, whereas the lymphocyte concentration decreases below pre-exercise levels during the post-exercise phase (7). The mechanisms behind the alterations in leukocyte counts post-exercise remain poorly understood. Significant attention has lately been directed into alterations in the distribution of lymphocyte subpopulations in response to exercise (8). There are two primary subpopulations of T lymphocytes that are distinguished by their surface proteins: CD4 + and CD8+. Although CD4 + cells are predominantly considered as inducing T-helper cells and CD8 + cells as cytotoxic/suppressive T inducer, both cell types possess intricate functions in the initiation and regulation of the inflammatory and immune response in humans and horses (7, 9). Engaging in physical exercise necessarily expends energy, produces reactive oxygen species (ROS), and stimulates the immune system, resulting in both beneficial and potentially detrimental effects contingent upon the kind and intensity of the immunological responses elicited. Regular engagement in moderate-intensity physical exercise in human is extensively recognized for fostering an anti-inflammatory state, hence preventing the onset of chronic diseases in the long term (10). The immune system’s activation leads to the secretion of cytokines, which can be categorized as pro-inflammatory (such as IL-1, IL-8, TNF-*α*, IFN-*γ*, etc.) or anti-inflammatory (including IL-2, IL-4, IL-10, IL-13) interleukins (11).

Cytokines are soluble proteins or glycoproteins, produced and secreted during inflammation, that mediate the communication between immune and non-immune cells and regulate biological processes and disease in humans (12). The production of cytokines upregulated rapidly in response to inflammatory stimuli, and this response can be transient or prolonged (1). Cytokines are poorly studied in horses and were known to function as physiological safeguards against tissue damage, injury, and illness (13–15). Cytokines constitute a complex array of signaling pathways that interact with the immunological and endocrine systems as part of the intricate response to many physiological stresses, including exercise (16). Moderate activity levels elicit anti-inflammatory mechanisms, while intense exercise levels activate pro-inflammatory pathways in the cytokine response to exercise (2). For example, a pro-inflammatory cytokine IL-1, exhibited a considerable elevation during high-intensity exercise in comparison to low-intensity exercise in human subjects (16, 17). This is vital for the long-term adaptive responses to exercise training which depend on the type, intensity, duration and familiarity of the exercise, as well as the age and clinical condition of the participants (1, 3, 18). Furthermore, moderate-intensity aerobic exercise during a four-week period resulted in a reduction of tumor necrosis factor-alpha (TNF-*α*) (19). Hence, high-intensity physical training or competitions have been demonstrated to induce a pro-inflammatory condition. Intense and prolonged exercise also induces temporary immunosuppression referred to as “the open window” theory that is widely discussed in humans. The “open-window” hypothesis posits that a decline in immune function following intense exercise heightens the susceptibility to infections in human athletes (20–22). The activation of the immune system is a reaction to a stressor, intended to re-establish cellular homeostasis. One possible cause is the change in redistribution of peripheral and central populations of white blood cells (WBC). The inflammatory process is essential for homeostasis primarily via proactive defence against diverse detrimental stressors, including neurotropic viral infections and/or traumatic injury, facilitating the restoration of cellular and tissue functionality (23). The innate (natural) and acquired (adaptive) immune systems are responsible for the primary defence against infections (24). Although numerous studies have been conducted in humans, few studies involving the equine athlete (25, 26) or dogs (27) have been conducted in this regard. Based on recent literature in horses, the effect of exercise on the physiological response and adaptation of the animal has been observed based on frequency, intensity, discipline, and duration of activity (28–31). Few considerations have been conducted about the optimal time of day for exercising in horses. The mechanisms underlying daily variations in equine athletic performance remain ambiguous and can be attributed to numerous factors, including habitual exercise timing, individual chronotype, sleep patterns, dietary intake, environmental conditions, and the endogenous circadian clock (32, 33). Whether the timing of training affects physical adaptations, it would be necessary to significantly revise training programs by incorporating the optimal time of day to perform physical activity. There is no evidence indicating an appropriate time of day for exercise concerning the body’s inflammatory reactions and daily variations in immunological indicators in horses (34–36).

Understanding the influence of time-of-day on horses’ physiological reactions to exercise and the mechanisms underlying chronoperformance can significantly impact the organization of training regimens, work routines, and competitive events. The objective of the present research was to examine the response of some inflammatory biomarkers (white blood cell count-WBCs, leukocyte subpopulation CD4 + and CD8 + cells, total proteins, Interleukins IL-6, IL-1β and Tumor Necrosis Factor TNFα) based on exercise performed in the morning (*am*) and afternoon (*pm*).

## 2 Materials and methods

### 2.1 Animals

All animal-handling procedures were conducted by the principles outlined in the Declaration of Helsinki, the directive 2010/63/EU of the European Parliament and of the Council of 22 September 2010 on the protection of animals used for scientific purposes and approved by the Ethical Committee of University of Messina (code: 06/2023).

The present study was conducted on ten Italian Saddle horses considering five non-pregnant, non-lactating mares and five geldings, aged from 10 to 15 years old, with a mean body weight of 500 ± 30 kg used after the signed informed consent from the owners. The physiological and clinical state of the horse was assessed by monitoring heart rate, respiration rate, rectal temperature, faecal consistency, and conducting haematological and haematochemical profiling. Animals with injuries or those beyond the physiological range for horses were eliminated from the study. The individuals were devoid of internal and external parasites, having had treatment every 3 months.

During the experimental protocol, the horses were housed in identical individual boxes of 3.5 × 3.5 m and 6 m in height at a private horse training centre in Sicily, Italy (Latitude 38° 7’ N; Longitude 13° 22′E). A multiparametric probe (Testo 400) was used to monitor the environmental parameters during the experimental period. Boxes were provided with a 1.5 × 1.5 m window, and a grid positioned in front of the box wall facilitated social interaction. The horses were fed with a specialized diet for trained adult equines, consisting of high-quality hay and concentrates (crude protein 16%, crude fat 6%, crude fibre 7.35%, ash 10.09%, sodium 0.46%, lysine 0.85%, methionine 0.35%, omega-3 0.65%). Water was accessible *ad libitum*. All animals were fed three times daily (6:30, 12:00 and 19:00) and received the same diet. This particular feeding schedule was previously shown in other trained athletic horses not to influence the AM and PM training session (37).

Training and animal care were conducted by experienced stable personnel unaffiliated with the research team.

### 2.2 Protocol

Each horse was tested on show jumping sessions in the morning *am* around 10:00 and in the afternoon *pm* around 16:00 (sunrise 05:05, sunset 20:45) in randomized order within the 48 h so that the two sessions conducted were not closely spaced. The hours of the day for the *am* and *pm* sessions were chosen based on the circadian pattern of the main immune and inflammatory parameters in horses (38), without interfering with the normal routine of the animal.

The experimental tests were performed in a 40 × 20 m indoor dirt-footing arena. The indoor facilities were chosen to have environmental conditions as much as possible constant throughout the test. The exercise included a warm-up, consisting of walking, trotting, and galloping and six jumps.

A systemic inflammatory response including enhance expression of leukocytes, TNFα, IL-1β and IL-6 after moderate physical exercise have already been discussed in horses, humans and rats. Thus, a moderate intensity training program was considered according to previous studies (39). The same rider (65 kg + 5 kg of tack) rode the horses in the morning and afternoon tests.

### 2.3 Sampling

Blood samples were collected by jugular venipuncture into 4-mL Vacutainer tubes (Terumo Corporation, Japan) containing ethylenediaminetetraacetic acid (EDTA) and without anticoagulant at different sampling times: before, immediately (following 5 min) and 1 h after the exercise during the morning (*am*) and afternoon (*pm*) training sessions. Serum was separated by centrifugation at 3000 r.p.m. for 10 min, placed in separate Eppendorf test tubes and stored at −20°C until analysis. A blood smear was performed from the EDTA tubes immediately after the blood collection for leukocyte (neutrophils, basophils, eosinophils, lymphocytes, and monocytes) population identification and counting. All blood samples were refrigerated at 4°C and analyzed for complete blood count within 2 h by means of an automated hematology analyzer (HeCo Vet C, SEAC, Florence, Italy).

### 2.4 Flow cytometry analysis

Flow cytometry technique was performed on EDTA samples for CD4 + and CD8 + cell subpopulation determination. Antibody standardization was carried out to find the minimum amount of antibody necessary to find the best MESF response (equivalent soluble fluorochrome molecules).

**Figure 1:**
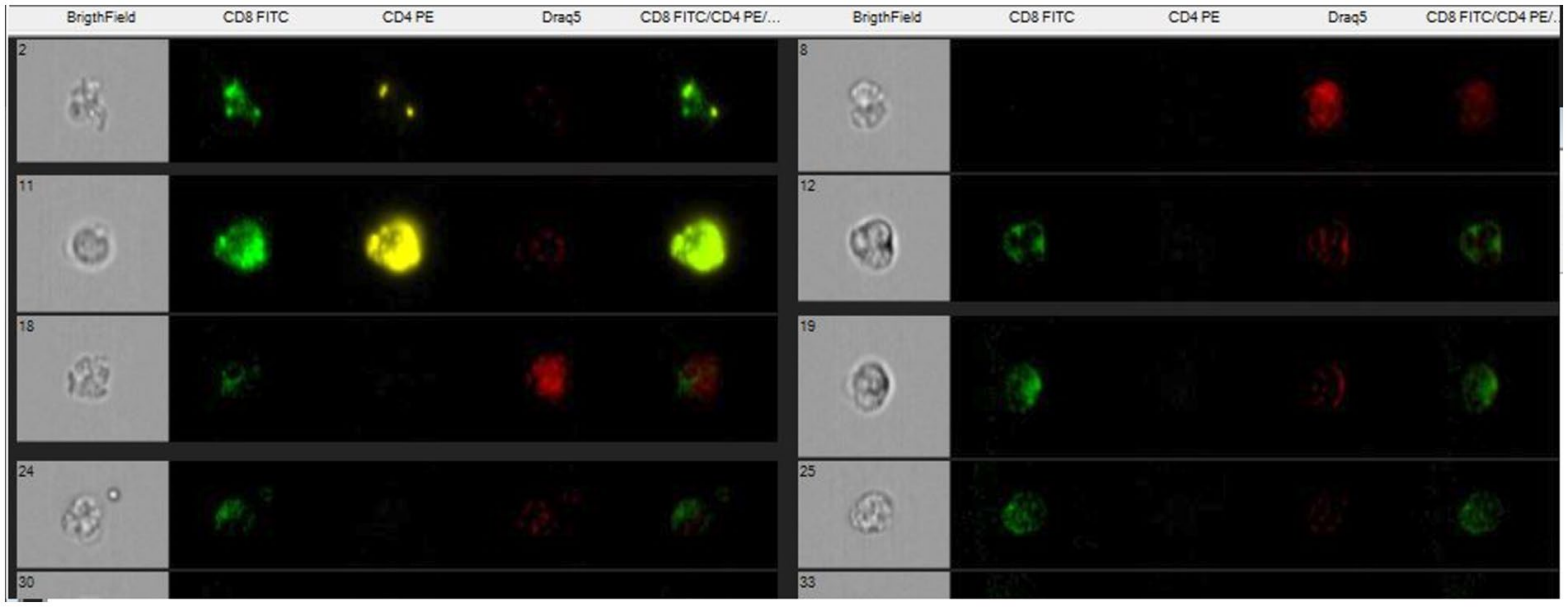
Scatter plots show recruitment of focused lymphocytes and the area where to find the marked cells with antibodies.

**Figure 2:**
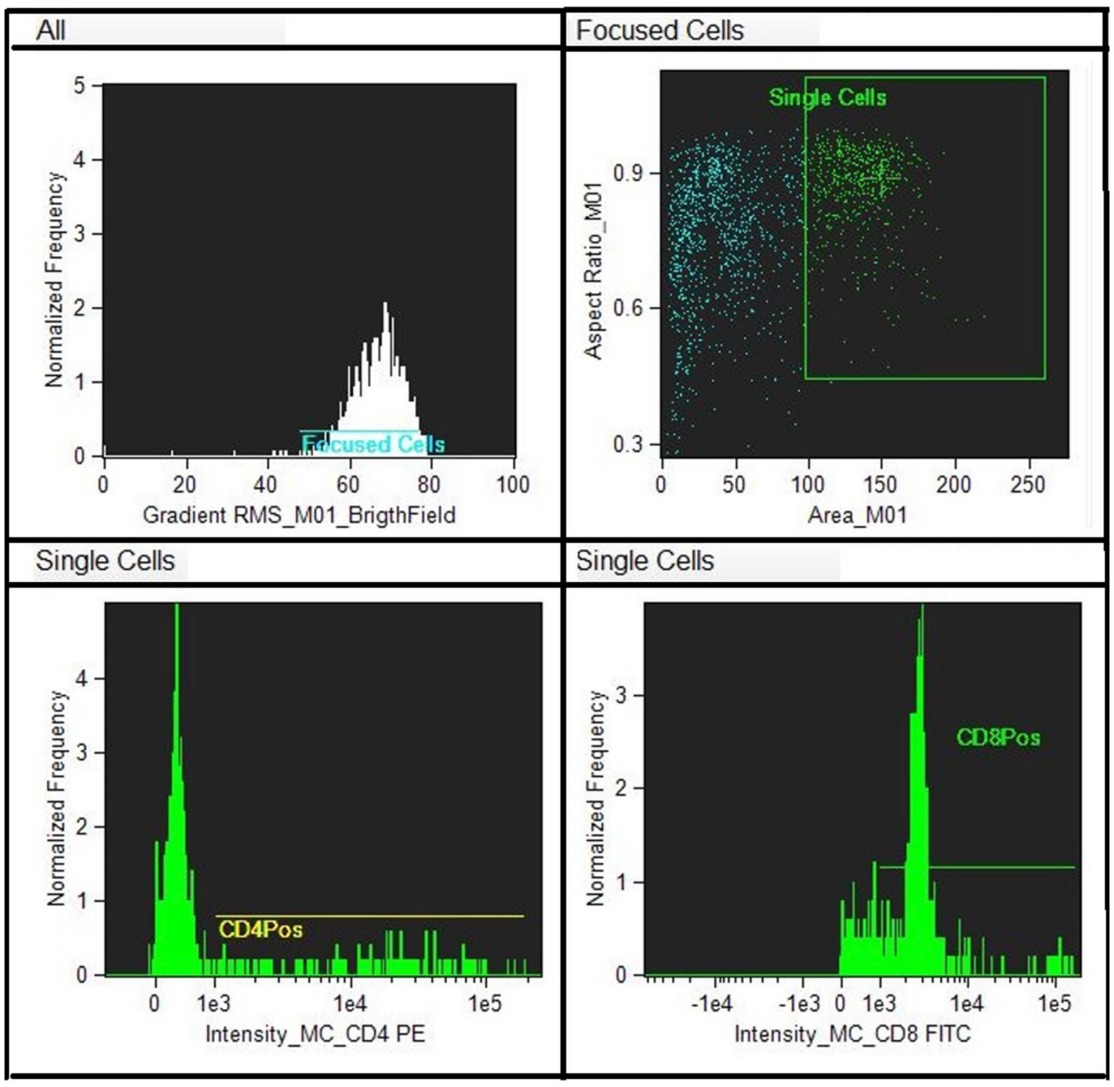
Single marked cells with CD8-Fict, CD4- PE and nucleus- Draq5 and unmarked.

A titration of the monoclonal antibody was performed to get the best result with the least amount of antibody (between 1:20 and 1:200). Monoclonal antibodies (Biorad) were used for the determination of the lymphocyte subpopulations, CD4 and CD8 conjugated with different fluorochromes (Fitc and PE). Draq5 (nuclear dye) was also used to stain the nucleus to provide better image contrast. The analysis was performed on the EDTA sample (100 μL of whole blood) to obtain biparametric assays on a single blood sample. The antibodies are reactive against the equine species. The CD8 clone CVS8 and the CD4 clone CVS21. EDTA samples (100 μL) was moved to empty tubes where 3 μL of respective markers were included. Samples in EDTA were initially incubated for 30′ in the dark to avoid fluorochrome degradation. The analytical method utilizing Image Stream does not require the lysis of red blood cells, as they are excluded through the gating analysis of lymphocyte populations. However, following incubation with monoclonal antibodies, the cells were lysed using an ammonium chloride solution and subsequently fixed in a 0.5% paraformaldehyde solution to enhance sample stability and performance. Flow cytometric analysis was performed using a multispectral flow cytometer -ImageStreamX (Amnis, Seattle, WA), in which standard microscopy was combined with flow cytometry. This instrument can acquire up to 100 cells/s, with the simultaneous acquisition of six images of each one, including multiple fluorescent images, bright field, and scatter. In addition, the integrated software INSPIRE, runs on the ImageStreamX Mark II. Each prepared sample was maintained on ice before to ran into the flow cell. Then, the cells were allowed to form a single core stream before acquisition. Images were analyzed using IDEAS image-analysis software (Amnis). Approximately 10,000 cells were acquired and analyzed to assess the percentage of CD4 and/or CD8-positive cells in the sample. The analysis of the CD4 + and CD8 + lymphocyte subpopulations was carried out within a pool of previously selected, focused and isolated leukocyte cells. The analysis technique does not, like classical flow cytometry, involve an actual gating strategy but a selection of images (gradient) of the clear field of positively fluorochrome- marked lymphocyte cells that are in focus. Of the images that are in focus, the size and appearance of individual cells are identified so that they can be showed at the graphical level as images. The control was internal as some cells are marked others are not as shown in Figures 1, 2. Within the selected areas, the cells superficially marked with CD4 + and those marked with CD8 + and nucleus were highlighted to better distinguish the internal from the superficially marked components of the individual cells observed, as shown in Figures 1, 2. The lymphocyte identification strategy involves the same use of the two monoclonal antibodies that identify the T population of lymphocytes. Compensation for image flow is made only by acquiring cells stained for single fluorochrome without side scatter. The cutoff between negative and positive is related to the high sensitivity of the experiment, so there is no need to use isotype control, which is rightly needed in classical flow cytometry.

### 2.5 Biochemical analysis

On obtained serum samples, the concentration of interleukins 1β, 6 and tumor necrosis factor-*α* (IL-1β, IL-6 and TNF-α) was assessed through enzyme-linked immunosorbent assay kits specific for equine species (Equine IL-1β ELISA kit, sensitivity 3.5 pg./mL; RayBio®, Equine TNF- α ELISA kit, sensitivity 0.64 pg./mL, RayBio®; Equine IL-6 ELISA kit, sensitivity 5.5 pg./mL; MyBiosource®) by means of a micro-well plate reader (Sirio, SEAC, Florence, Italy). For all used tests, Intra-Assay CV% was < 10%, and Inter-Assay CV% was <12% determining reproducibility. All calibrators and samples were run in duplicate, and samples exhibited parallel displacement to the standard curve for each ELISA analysis. The concentration of IL1β, IL-6, and TNFα were obtained from the resulting optical densities (OD) used for calculating each standard curve.

### 2.6 Statistical analysis

Data were normally distributed (Kolmogorov–Smirnov test *p* > 0.05) and reported as mean ± standard deviation (SD). Two-way for repeated measure analysis of variance ANOVA was applied to each parameter recorded during the experiment to establish the effect of exercise (before vs. immediately and vs. 1 h after exercise) and the effect of time of day (*am* vs. *pm*). Bonferroni’s test was applied to perform a *post hoc* comparison. Differences were considered statistically significant at *p* < 0.05. Data were analyzed using statistical software Prism v. 9.00 (Graphpad Software Ldt, Solana Beach, CA, USA).

## 3 Results

The results were expressed as mean ±Standard Deviation SD. The environmental temperature and relative humidity during the *am* hours were 25.5 ± 0.07°C and 49.5 ± 0.5% respectively, and 24.7 ± 0.08°C and 61.1 ± 0.5% in the *pm* hours. Two-way for repeated measure ANOVA showed a significant effect of exercise on WBCs, CD4 + and IL-6, and of time of day (*am* vs. *pm*) on CD4+, CD8+, IL-1β and IL-6. No significant variation was observed for leukocyte subpopulation, total proteins and TNF-*α* during the exercise sections. The application of Bonferroni’s *post hoc* comparison showed an increase of WBCs immediately and 1 h after exercise (*p* < 0.01: *p* < 0.01) compared to the rest value at *am*. On the contrary, significant decreasing values of CD4 + were observed immediately and 1 h after exercise (*p* < 0.01; *p* < 0.01) compared to rest at *pm* as shown in Table 1. Moreover, CD4 + cells showed significant lower values 1 h after exercise at *pm* compared to *am* (*p* < 0.001). CD8 + showed a significant lower value at *pm* compared to *am* for each time point (before: *p* < 0.01; immediately: *p* < 0.01; 1 h after: *p* < 0.001). IL-6 showed a significant increasing concentration after exercise at *pm* (*p* < 0.01). IL-6 and IL-1β were significantly higher at *pm* compared to *am* at each time point (*p* < 0.0001; *p* < 0.0001; *p* < 0.0001) as shown in Table 2.

**Table 1:** Mean± standard deviation SD of CD4+, CD8+, WBC, neutrophils, lymphocites, monocytes, eosinophils and basophils expressed in their conventional units recorded before, immediately and 1 h after exercise in the *am* and *pm* sessions together with their statistical significances observed.

Experimental conditions			CD4 + %	CD8 + %	WBC x1012/L	Neutrophils %	Lymphocites %	Monocytes %	Eosinophils %	Basophils %
AM	Before	Mean	23.3	10.3	4.8	54.0	40.5	2.0	0	0.7
		SD	5.7	2.13	0.5	4.32	6.4	0	0	1.1
	After	Mean	20.3	12.5	6.7^A^	58.0	39.0	2.2	0.7	0.3
		SD	4.3	3.8	1.8	6.3	7.0	0.5	1.1	0.6
	1 h after	Mean	22.2	11.3	6.7^A^	55.0	42.2	1.2	1.0	0.3
		SD	8.5	5.2	1.8	3.5	5.4	0.9	0.6	0.6
PM	Before	Mean	21.7	6.7*	5.5	60.2	34.7	2.0	0	0.3
		SD	3.0	0.6	0.9	5.5	3.8	0.8	0	0.5
	After	Mean	18.9^A^	7.3*	5.5	62.5	35.5	1.7	0.3	0
		SD	4.2	2.1	0.2	5.7	4.4	0	0.5	0
	1 h after	Mean	13.5^A^*	4.4*	6.5	58.5	36.0	2.2	0.3	1.0
		SD	6.7	1.2	1.5	5.9	3.6	0	0.6	0.8

Capital letters (A vs. before) indicate the effect of exercise and symbol (* vs. *am*) indicate the effect of time of day.

**Table 2:** Mean± standard deviation SD of IL-6, IL-1β, TNFα expressed in their conventional units recorded before, immediately and 1 h after exercise in the *am* and *pm* sessions together with their statistical significances observed.

Experimental conditions			IL-6 pg./mL	IL-1β pg./mL	TNFα pg./mL
AM	Before	Mean	1.78	1.63	1.08
		SD	0.50	0.65	0.32
	After	Mean	2.0	1.71	1.12
		SD	0.76	0.79	0.37
	1 h after	Mean	1.87	1.66	1.14
		SD	0.75	0.66	0.41
PM	Before	Mean	1.89*	1.96*	1.17
		SD	0.74	0.65	0.38
	After	Mean	2.53^A^*	1.92*	1.16
		SD	1.34	0.65	0.38
	1 h after	Mean	1.86*	1.94*	1.17
		SD	0.81	0.66	0.37

Capital letters (A vs. before) indicate the effect of exercise and symbol (* vs. *am*) indicate the effect of time of day.

## 4 Discussion

Physical exercise during sport training is known to influence blood parameters (40). The present study showed the influence of exercise and time of day on certain inflammatory biomarkers in athletic horses. In particular, the current findings indicated a notable elevation in WBC levels at *am.* Literature indicates that elevated WBC counts are among the most constant physiological reactions to exercise, often observed following all forms of physical activity. Exercise-induced leukocytosis is frequently compared to an inflammatory response, with the increase being associated with the immune system’s reactivity to physiological stressors (24). Exercise-induced leukocytosis may be classified as pseudoleukocytosis since it does not stem from the generation of new cells, but rather from an elevation in lymphocyte count due to heightened adrenaline secretion, facilitating their release into the bloodstream from the spleen and, to a lesser degree, from the bone marrow and lymph nodes, explaining the increase observed only during the morning session (41).

Exercise is also known to affect lymphocyte subpopulations in horses (42, 43). A decreasing value in CD4 + levels was also observed in the present findings as observed by previous studies in racehorses. These values, based on their suppressive function depends on intensity and fitness of training and could possibly be related to the lymphocyte redistribution to peripheral tissues (44–46). Numerous contentious viewpoints exist about CD4 + and CD8 + behavior in horses and human post-exercise (43, 47). Previous studies generally indicate that exercise-induced reductions in lymphocyte subsets are transient and contingent upon exercise intensity and duration; specifically, high-intensity exercise tends to decrease the number of CD4 + cells in horses (42). Furthermore, the examined lymphocyte subpopulations CD4 + and CD8 + exhibited elevated levels in the morning compared to the afternoon at each time point. The fact that the difference between morning and afternoon is also observed in basal value, suggests that there is not an effect of exercise as much as it is related to daily changes in parameters, considering the natural peak of daily oscillations observed for CD4 + during the afternoon hours (38, 48).

The response to exercise in horses did not affect total proteins, as observed by previous findings (49). Consequently, the little variations noted between morning and afternoon during exercise may be more accurately ascribed to daily oscillations of the parameters (36, 39). Physical activity and its physiological effects necessitate a coordinated cytokine response, as these molecules are the primary regulators of immunological pathways. Exercise induces trauma in muscles, resulting in metabolic exhaustion and tissue damage, which elicits an inflammatory response. The current findings indicated a rise in IL-6 concentration following afternoon exercise, in contrast to morning exercise, during which other interleukin levels remained stable. Numerous studies indicate that the cytokine profile serves as an effective biomarker for assessing an athlete’s performance, recovery during training, and overall well-being in both horses and humans (50, 51). Cytokines in horses exhibit a specific action in the blood substrate, regarded as a direct delayed response to muscle injury and inflammation resulting from physical exertion (43). The cytokine response to training in horses may fluctuate based on variables including the type, intensity, and duration of exercise, as well as individual variables and additional factors (52). As already known IL-6 is a pleiotropic cytokine with dual anti-inflammatory and pro-inflammatory role (20). Its increase observed in post-exercise and no variation in other interleukins might emphasize the anti-inflammatory action of IL-6, as opposed to the pro-inflammatory action of IL-1*β* and TNFα (53). This increase could indicate an important strategy the body put into practice in order to limit the pro-inflammatory reaction due to exercise-induced muscle damage as previously observed in other studies (54, 55). A markedly elevated levels of IL-6 and IL-1 β were noted in the afternoon compared to the morning at each time interval. This significant increase might indicate an effect more related to circadian fluctuations of the following parameters than exercise-related changes (55). Therefore, on the one hand, the type of exercise performed in the afternoon could have a protective action due to the increase in IL-6 but on the other hand, exercise performed in the morning could be more optimal since it does not stimulate a significant production of interleukins, which are already in themselves regulated at the circadian level (55). The present study revealed limitations such as the small sample size, potential confounders that may have influenced the present results (feed timing, daily humidity differences, time between exams, horse fitness) and the lack of a control group that was not undergoing exercise to be compared.

## 5 Conclusion

The present study, conducted on athletic horses, showed an effect of both physical exercise and time of day on some of the parameters analyzed. Specifically, the effect of exercise observed both in the morning and evening does not seem to be attributable to the moderate intensity of exercise itself, but rather to its possible natural daily variations, which can also be observed from basal values. Based on this leukocytes and lymphocyte populations rise in the morning or remain stable with high values, while in the afternoon hours they tend to fall or remain constant. The same could be observed for the pro-inflammatory cytokine response that have lower values in the morning. This leads to an appreciation of the role of the immune system and the inflammatory response during exercise performed in the morning. This information is critical for the development of appropriate training regimens, considering performance in relation to a potential period of risk, during the day, for infection, disease and inflammation to which the horse may be susceptible. Based on the potential risk period fostered by physical stress, understanding the chronoperformance of the athletic horse could facilitate the identification of the best time of day for daily training.

## Data Availability

The original contributions presented in the study are included in the article/supplementary material, further inquiries can be directed to the corresponding author.
